# Non-Hodgkin's Vaginal Lymphoma: A Rare Presentation

**DOI:** 10.7759/cureus.28364

**Published:** 2022-08-24

**Authors:** Asma Usman, Anum Sultan, Sara Rehman, Anis Rehman

**Affiliations:** 1 Radiology, Shaukat Khanum Memorial Cancer Hospital and Research Centre, Lahore, PAK

**Keywords:** mri, pet ct, chemotherapy, immunohistochemistry and biopsy, vaginal lymphoma

## Abstract

Vaginal non-Hodgkin's lymphoma (NHL) is a rare entity. We report a case of a 38-year-old lady who presented with complaints of lower abdominal pain for three to four months and vaginal bleeding for one month. Her examination under general anesthesia revealed a hard vaginal mass which was biopsied and immunohistochemistry was performed. Diagnosis of diffuse B‑cell NHL (DLBCL) was made. Imaging plays an important role to reach the diagnosis. Chemotherapy is the treatment of choice.

## Introduction

Non-Hodgkin's lymphoma (NHL) involving the female genital tract as the primary site is a very uncommon presentation. Research from the National Cancer Database shows that 2% of extra-nodal NHL arises in the female genital tract [1]. Its rarity is ascertained by the fact that out of 9500 women with malignant lymphoma, only four are vaginal in origin [2]. 

Imaging plays a vital role to reach the diagnosis of vaginal lymphoma. MRI pelvis shows the exact extent of disease and its relation with adjacent viscera. CT abdomen and pelvis can help to define the regions of disease involvement and nodal stations. Recently, positron emission tomography-computed tomography (PET-CT) is the imaging modality of choice as it shows more accurately the stage of the metabolically avid disease. PET-CT is also helpful to see disease regression/progression and recurrence [3]. Surgery does not play any role as chemotherapy is relatively more effective [4]. 

The main purpose of this case report is to alert doctors to pay attention to such rare entities so that the patients presenting with non-specific vague symptoms could be evaluated timely and treated properly. Consideration of a wide range of differential diagnoses in female patients with vaginal solid mass should also be given. Moreover, it guides the clinicians to choose the correct imaging modalities which can further help in making the diagnosis.

## Case presentation

After obtaining the patient's informed consent and approval from the ethical review board, we present a case of a 38-year-old lady, known diabetic, referred to the radiology department with complaints of three to four months history of lower abdominal pain and per vaginal bleeding for one month. There were no associated 'B' symptoms such as fever, tiredness, and night sweats. She had a history of having irregular menstrual cycles since menarche. Her baseline laboratory investigations are as follows in Table 1.

**Table 1:** Baseline investigations WBC: white blood cell; RBC: red blood cell; MCV: mean corpuscular volume; MCH: mean corpuscular hemoglobin; MCHC: mean corpuscular hemoglobin concentration; RWD-CV: red cell distribution width - coefficient of variation; LDH: lactate dehydrogenase; ESR: erythrocyte sedimentation rate; GFR: glomerular filtration rate; HBsAg: hepatitis B surface antigen; HCV: hepatitis C virus; HIV: human immunodeficiency virus.

Investigations	Results	Units	Normal range
Complete Blood picture			
WBC	8.7	10 ^3^/ul	4-10
RBC	4.72	10 ^6^/uL	3.8-4.8
Hemoglobin	12.2	g/dL	12-15
Haematocrit	36.7	%	36-46
MCV	77.8	fL	76-96
MCHC	33.2	g/dL	31.5-34.5
MCH	25.8	pg	27-32
%RDW-CV	14.7	%	11.5-14.5
Platelets	262	10^3^/uL	150-450
Neutrophils	73.3	%	40-80
Lymphocytes	15.7	%	20-40
Monocytes	9.6	%	2-10
Eosinophils	1.3	%	1-6
Basophils	0.1	%	<1
ESR	4.1	mm/1^st^ hour	0-20
LDH	294	U/L	135-214
Glycohemoglobin (HbA1C)	9.4	%	5.7
Serum Electrolytes			
Sodium	135	mmol/L	136-145
Potassium	4.2	mmol/L	3.5 -5.1
Chloride	99.2	mmol/L	98-107
Bicarbonate	22.5	mmol/L	22-29
Urea nitrogen	11.32	mg/dL	6-20
Creatinine	0.66	mg/dL	0.70-1.20
eGFR	100.72	mL/min/1.73 m^2^.	<60
HBsAg	Non-Reactive		
Anti-HCV	Non-Reactive		
Anti- HIV	Non-Reactive		
Anti-Hepatitis B core total (IgM and IgG )	Reactive		

Her anti-hepatitis B (HB) core total (IgG and IgM) was reactive for which Entecavir was given. Her renal and liver function tests were within normal limits.

An examination under anesthesia was performed which demonstrated normal external genitalia. A firm tumorous growth was noted extending from the vaginal apex up to the introitus near the hymenal ring also involving the left vaginal wall. A band-like growth was noted on the right vaginal wall as well. Cervix was not visualized. The rectal exam was normal. Biopsy of vaginal mass, left and right vaginal walls were taken and an extensive panel of immunohistochemical staining was performed.

Immunohistochemistry picture showed CD 20 positive in lymphoma cells, PAX5 positive in some lymphoma cells, Ki 67 50-60% proliferation index, and negative CD 10 and CD 5 immunohistochemical stains. Diagnosis of B-cell non-Hodgkin's lymphoma likely diffuse large B-cell lymphoma was made.

**Figure 1:**
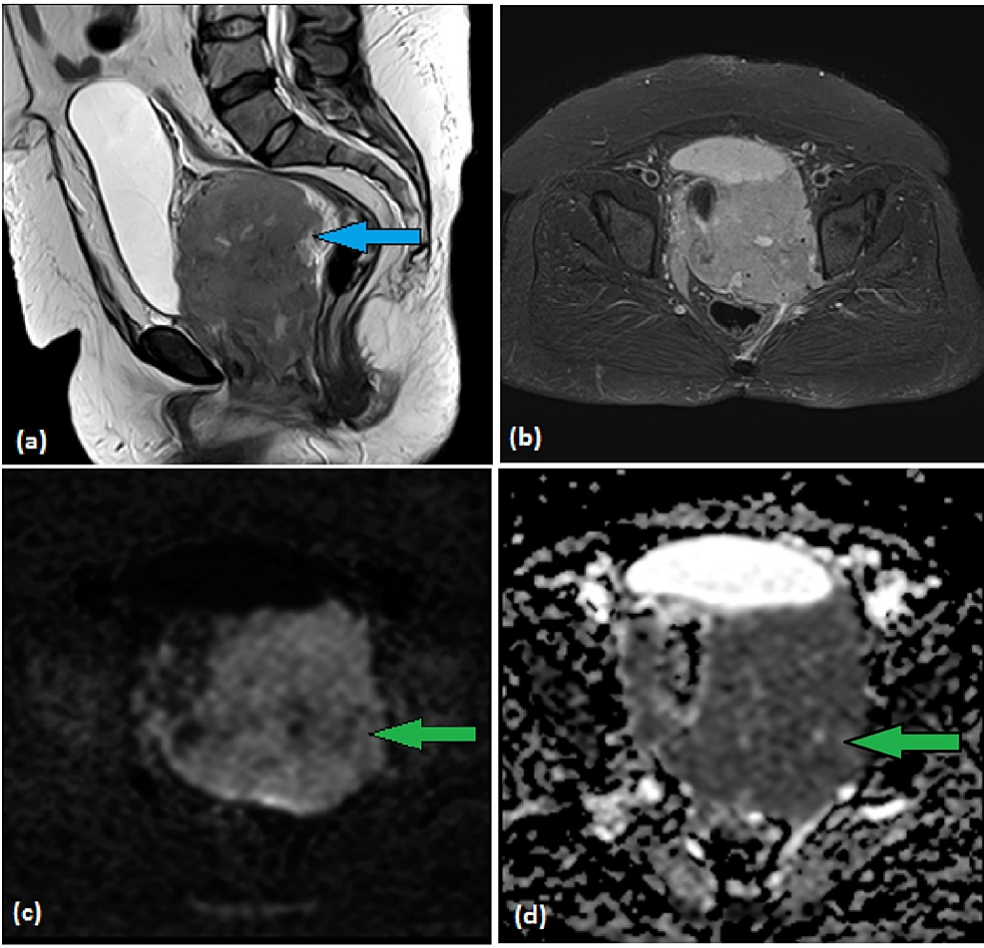
MRI T2W sagittal (a), axial STIR (b), DWI (c), and ADC (d) images demonstrating large vaginal mass showing low signals on T2W (blue arrow) and high signals with diffusion restriction (green arrows). T2W: T2 weighted, STIR: Short tau inversion recovery, DWI: Diffusion-weighted imaging, ADC: Apparent diffusion coefficient

A pelvic MRI was performed that shows a large infiltrative predominantly vaginal/uterine cervix mass with parametrical, pelvic sidewall, and bladder infiltration, entrapping both ureters with the resultant hydro ureter, right pelvic side wall lymphadenopathy and a thin layer of pelvic ascites concerning for disseminated peritoneal disease (Figure 1).

**Figure 2:**
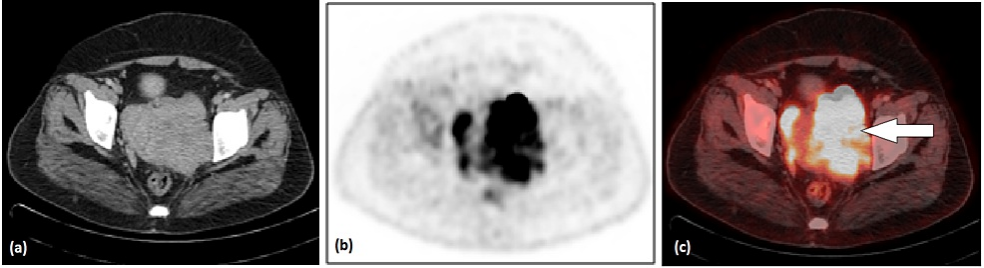
Pelvic CT (a), PET (b) and PET CT fusion (c) axial images showing hypermetabolic cervical/upper vaginal mass (white arrow) with parametrial extension and left pelvic sidewall involvement.

Her baseline 18F-fluorodeoxyglucose (18F-FDG) PET-CT was performed in January 2021 which showed hypermetabolic lobulated enhancing soft tissue mass centered at cervix/upper vagina measuring 11 x 10 cm with SUVmax of 7.4. The mass is causing a significant local effect on the uterus and urinary bladder. It is infiltrating into the parametrium, left pelvic sidewall, and vaginal canal (Figure 2).

The patient was diagnosed as having stage 4AEX (vaginal/uterine/cervix mass, pelvic sidewall, and bladder infiltration) disease on imaging according to the Ann Arbor classification system used for staging of NHL [5]. She was started on chemotherapy using RCHOP (rituximab, cyclophosphamide, hydroxydaunorubicin hydrochloride (doxorubicin hydrochloride), vincristine (Oncovin), and prednisone) with three cycles given up to May 2021.

**Figure 3:**
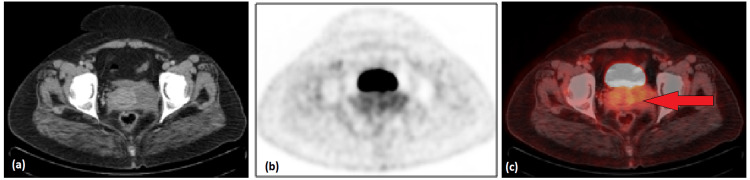
Interim pelvic CT (a), PET (b) and PET CT fusion (c) axial images showing good partial treatment response with interval regression in size and metabolic activity of the cervix/vaginal mass (red arrow).

Interim PET-CT after three cycles of chemotherapy in April 2021 showed an interval decrease in size and metabolic activity of cervix/upper vaginal mass measuring 8.4 x 7.0cm with SUVmax of 4.9 without any local compression effect (Figure 3).

**Figure 4:**
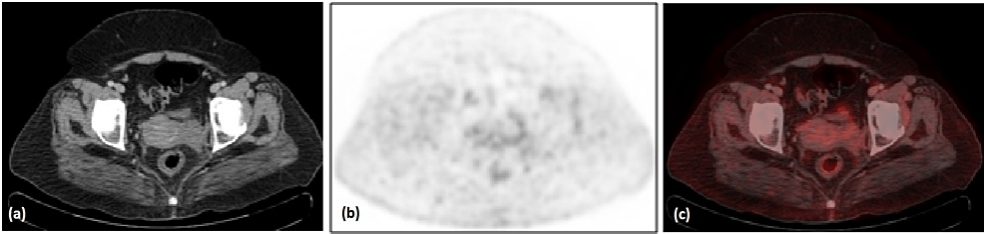
End of treatment pelvic CT (a), PET (b) and PET CT fusion (c) axial images showing complete metabolic response.

Following the six cycles of chemotherapy, her end-of-treatment PET-CT scan was performed in November 2021 which showed a complete metabolic response with residual non-FDG avid soft tissue fullness in the left adnexa (Figure 4).

## Discussion

NHLs are known lymphoid neoplasms, having a separate morphologic, immunophenotypic, and genetic constitution. NHLs of the genital tract are rare, usually affecting females between the ages of 26 to 66 years [6]. The cervix is the most common site of involvement followed by the ovaries and the uterus [6]. The usual presentation is a hard bulging mass with vaginal bleeding [6].

Malignant vaginal masses include a wide range of differential diagnoses such as carcinomas, NHL, malignant Mullerian masses, leiomyosarcoma, and stromal masses [7]. Accurate diagnosis can only be established with biopsy and histopathological correlation along with the use of an extensive panel of immunohistochemical stains. It is very important to differentiate whether the tumor is CD 20 specific for B cells, CD 3+ specific for T cells, or CD 45 + specific for granulocytic sarcoma to differentiate between different tumors of the female genital tract [8]. On histopathology, DLBCL has specific morphology and immunochemistry staining of tumor cells. These tumor cells are large B cells with large nucleoli, basophilic cytoplasm, and a unique cell growth pattern with an increased proliferation rate. Pan-B cell antigens are present on tumor cells which are CD 19, CD 20, CD 22, and CD 79a positive [8]. CD 10 is always negative in NHL. These stains are an integral part of the diagnosis of non-Hodgkin's lymphoma.

As tumor cells are highly responsive to chemotherapy, it becomes the main and important treatment for NHL [9]. For early stages of DLBCL, first-line chemotherapy includes CHOP-R, including cyclophosphamide, doxorubicin, vincristine, prednisone, and rituximab, a monoclonal antibody directed against the CD 20 antigen. If proper and timely treatment of primary pelvic lymphomas was initiated, then a five-year survival rate of 85% to 91% is reported [10]. Our case shows a complete metabolic response with good morphological treatment response to chemotherapy.

It is very important to know about factors that can help to reach the diagnosis of NHL earlier to have good results. MRI findings of significantly restricted diffusion with lower apparent diffusion coefficient (ADC) help to differentiate vaginal NHL from carcinomas. MRI also helps to exclude recurrence of the disease. Other vaginal masses also look like lymphoma. The following are a few differentiating points: carcinosarcoma shows intense enhancement on MRI post-contrast equal to the enhancement of myometrium and appears hyperintense on T2-weighted images; leiomyosarcoma shows internal blood components and appears high on T1-weighted images; endometrial stromal sarcomas are very infiltrative involving parapelvic sidewalls and show mixed signal intensity on T2-weighted images due to hemorrhage; and squamous cell carcinoma of the vagina is isointense to muscle on T1-weighted images and shows an intermediate signal on T2-weighted images [11].

PET-CT is helpful, especially for staging purposes and identifying distant metastasis [12]. It has also established a role in response evaluation as well as in disease surveillance [5,12]. Baseline imaging is important for comparison to see the response of therapy; these findings are important in the context of NHL to start chemotherapy. 

## Conclusions

Vaginal primary NHL is a rare entity. Patients should benefit from advanced imaging techniques such as MRI and PET-CT by having an early diagnosis and initiating early treatment of choice. The following case report will help the doctors to think about the differential diagnosis of vaginal masses. Chemotherapy is a magical therapy for vaginal NHL.
